# Multi-biobank summary data Mendelian randomisation does not support a causal effect of IL-6 signalling on risk of pulmonary arterial hypertension

**DOI:** 10.1183/13993003.02031-2023

**Published:** 2024-04-04

**Authors:** Benjamin Woolf, James A. Perry, Charles C. Hong, Martin R. Wilkins, Mark Toshner, Dipender Gill, Stephen Burgess, Christopher J. Rhodes

**Affiliations:** 1 The MRC Integrative Epidemiology Unit, University of Bristol, Bristol, UK; 2 School of Psychological Science, University of Bristol, Bristol, UK; 3 The MRC Biostatistics Unit, University of Cambridge, Cambridge, UK; 4 Department of Medicine, University of Maryland School of Medicine, Baltimore, MD, USA; 5 Department of Medicine, Michigan State University College of Human Medicine, East Lansing, MI, USA; 6 National Heart and Lung Institute, Imperial College London, London, UK; 7 Department of Medicine, University of Cambridge, Cambridge, UK; 8 Department of Epidemiology and Biostatistics, School of Public Health, Imperial College London, London, UK; 9 British Heart Foundation Cardiovascular Epidemiology Unit, Department of Public Health and Primary Care, University of Cambridge, Cambridge, UK

## Abstract

Interleukin (IL)-6 has been linked with the pathobiology of pulmonary arterial hypertension (PAH). IL-6 plasma levels are elevated in PAH patients and closely linked to survival [1]. Both increased IL-6 activity and gene knockout influence the development of, and resistance to, pulmonary hypertension in animal models [2–4]. IL-6 can repress expression of *BMPR2,* a gene key in PAH risk [5].

## Shareable abstract

**In the most comprehensive analysis to date, this study failed to detect an association of genetically predicted CRP-weighted IL-6 signalling or CRP-weighted IL-6R signalling with PAH risk using all available PAH GWAS data** https://bit.ly/3T5h5uj

*To the Editor*:

Interleukin (IL)-6 has been linked with the pathobiology of pulmonary arterial hypertension (PAH). IL-6 plasma levels are elevated in PAH patients and closely linked to survival [1]. Both increased IL-6 activity and gene knockout influence the development of, and resistance to, pulmonary hypertension in animal models [2–4]. IL-6 can repress expression of *BMPR2,* a gene key in PAH risk [5].

Evidence for a direct role for IL-6 in PAH was investigated in an unblinded phase 2 clinical trial of the IL-6 receptor (IL-6R) antagonist, tocilizumab, in 19 idiopathic/heritable PAH patients and 10 connective tissue disease-associated PAH patients. Despite decreasing C-reactive protein (CRP) and increasing plasma IL-6 levels, treatment had no effect on pulmonary vascular resistance at 6 months [6].

The random inheritance of genetic variants associated with an exposure (such as a perturbation of a drug target such as IL-6 signalling) can be used to assess causal relationships with a phenotype (*i.e.* PAH risk) in a Mendelian randomisation (MR) study. If variants can only affect PAH risk *via* IL-6 signalling, then an association between the variants and PAH risk can be interpreted as evidence of a causal relationship. The variant–exposure genetic association does not need to be measured in the same study as variant–outcome associations, provided they have been sampled from similar populations. When multiple published genome-wide association studies (GWASs) estimate the same variant–outcome association, estimates from these GWASs can be combined by meta-analysis. Likewise, multiple independent variants are analogous to separate trials, so are typically also meta-analysed. The insight offered by MR studies is therefore limited by the number of known genetic predictors of the exposure, and power of outcome GWASs.

After the completion of an international GWAS for PAH, with over 2000 PAH cases confirmed by expert clinical centres [7], it became feasible to test the effect of IL-6 signalling on PAH risk using MR. Consistent with the lack of effect on pulmonary vascular resistance in the trial, a MR analysis showed no association between genetically predicted levels of IL-6R and PAH risk [6]. This study used the lead *cis* variant associated with plasma IL-6R protein measurements, which has previously been shown to mimic treatment with tocilizumab [8].

An alternative to only using the lead *IL6R* variant is to use multiple independent variants in the *IL6R* gene that are significantly associated with CRP levels, a downstream biomarker of IL-6 signalling. Although CRP is a non-specific inflammatory marker, genetically predicted CRP using variants solely from the *IL6R* gene should proxy the impact of interventions on IL-6 signalling. This approach should boost power relative to using a single variant. Zhang *et al.* [9] used six CRP-associated variants from the *IL6R* region with GWAS data derived from the Finnish population study (FinnGen round 5) to find an inverse association between genetic predictors of increased IL-6 signalling and risk of PAH.

Given the disparity in findings between the two studies, we used all available PAH GWAS data to explore if IL-6 signalling, proxied by CRP, is associated with PAH risk using variants from the *IL6R* gene region, and thus if IL-6R antagonists can be expected to impact PAH risk.

To maximise our power to detect an association we extracted summary data from all publicly available genetic studies containing PAH patients: 1) a 2019 meta-analysis of four international case–control studies of PAH, with 2085 cases and 9659 controls, all of European ancestry [7]; 2) a GWAS of PAH in the UK Biobank using 493 medical record-identified cases and 24 650 age-, sex- and ancestry-matched controls [10]; and 3) FinnGen round 9 (n=377 277), including 234 individuals with medical record-identified PAH (ID: I9_HYPTENSPUL) [11]. FinnGen does not overlap with either the UK Biobank or the 2019 meta-analysis. However, some UK Biobank cases could have independently volunteered for a participating cohort in the 2019 meta-analysis. Not accounting for participant overlap, if existent, will produce overly precise estimates.

As a biomarker for IL-6 signalling, we used blood CRP measurements, taken exclusively from 361 194 UK Biobank participants through a blood assay [12].

We used single nucleotide polymorphisms (SNPs) within a 100-kb window of the *IL6R* gene region (Chr1:154377669-154441926) that were associated with CRP levels. A p<5×10^−5^ threshold was chosen as the Bonferroni correction for the approximately 1000 SNPs available in the UK Biobank within the *IL6R* gene region.

We clumped SNPs so that they have pairwise correlation (r^2^) of at most 0.1 estimated using data from the 1000 Genomes Project provided in the TwoSampleMR R package [13]. After clumping, we included 12 SNPs as instruments. These SNPs had a mean F-statistic of 92 in the UK Biobank, the variants are therefore sufficiently strongly associated with CRP to give meaningful MR estimates. The variants explain 1% of the variance of IL-6 levels, implying that to achieve a given level of power an MR study will need a sample size 100 times larger than an observational study. We used the False discovery rate Inverse Quantile Transformation Winner's Curse correction to adjust for Winner's Curse bias [14]. We harmonised GWAS using the TwoSampleMR R package, and excluded palindromic SNPs that could not be aligned using allele frequencies [13].

Our MR estimator is an inverse variance weighted meta-analysis of the ratio of the variant–outcome association (derived from each PAH GWAS) to the variant–exposure association (derived from the UK Biobank) for each SNP. We did not account for weak correlation between variants to minimise the chance of missing a true effect.

After combining data from all three PAH GWAS, we did not find evidence for an association of genetically predicted CRP-weighted IL-6 signalling with PAH risk (OR (per mg·L^−1^ increase in CRP) 1.01, 95% CI 0.84 to 1.22) (figure 1). There was no indication of between-dataset or between-variant heterogeneity in our analysis (figure 1).

**FIGURE 1:**
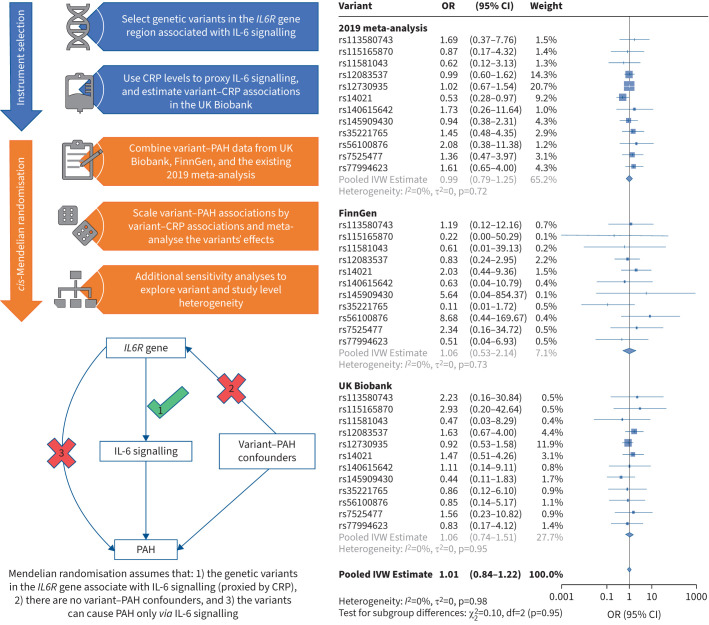
Study overview and main results showing associations of C-reactive protein (CRP) weighted genetically proxied interleukin (IL)-6 signalling with pulmonary arterial hypertension (PAH) risk for each genetic variant stratified by genome-wide association study (GWAS) data source. Odds ratios represent the multiplicative increase in the odds of PAH per mg·L^−1^ increase of genetically proxied CRP. In summary data Mendelian randomisation (MR) analyses, the effect of the exposure (CRP) on the outcome (PAH risk) is estimated by scaling the variant–outcome association (estimated in FinnGen, UK Biobank, and a 2019 meta-analysis) by the variant–exposure association (only estimated in the UK Biobank). The “rs” followed by a set of numbers (*e.g.* rs12730935) in this figure represent the variant-specific MR estimates from each GWAS for the 12 *IL6R* variants included in our analysis. The estimates of the “pooled IVW estimate” in grey is inverse variance weighted (IVW) meta-analysis of each GWAS's variant-specific effects. The estimates of the “pooled IVW estimate” in black is an IVW meta-analysis of the GWAS-specific effects.

Our null finding contrasts with Zhang *et al.* [9], who found that greater genetically predicted CRP-weighted IL-6 signalling was negatively associated with PAH (OR (per log unit increase in CRP) 0.023, 95% CI 0.001 to 0.393) using six genome-wide significant SNPs from the *IL6R* gene region in FinnGen round 5. While the FinnGen round 5 GWAS was large (n=162 959), there were only 125 medical record-identified PAH patients. It is typical to have more controls than cases in a disease GWAS, but beyond a 4:1 ratio further controls add negligible additional power [15]. Our updated analysis has greater than 20 times more cases than the study reported by Zhang *et al.* [9], and twice the number of variants. It should therefore be better powered than their analysis. That our analysis, even for FinnGen round 9 alone, is consistently null, suggests the non-null finding by Zhang *et al.* [9] is a false-positive finding.

In the most comprehensive analysis to date, we have failed to detect an association between genetically predicted CRP-weighted IL-6 signalling and PAH risk using all available PAH GWAS data and gene region-wide significant variants in the *IL6R* gene. This implies that IL-6 signalling may not be an important cause of PAH in unstratified populations. However, even with the large sample size, an important limitation of our analysis is very limited power and the resulting wide confidence interval which is compatible with a moderate effect for every mg·L^−1^ change in genetically proxied CRP.

## Shareable PDF

10.1183/13993003.02031-2023.Shareable1 This one-page PDF can be shared freely online.
Shareable PDF ERJ-02031-2023.Shareable

## Data Availability

statement: International PAH GWAS data are deposited to GWAS Catalogue and FinnGen summary statistics are available from https://www.finngen.fi/en/access_results. The data (*i.e*. variant-exposure and variant-outcome association summary statistics) and R code used in this study are available from https://doi.org/10.17605/OSF.IO/PZGE2.
