# Supplementary material for: Multi-biobank summary data Mendelian randomisation does not support a causal effect of IL-6 signalling on risk of pulmonary arterial hypertension
Source: Eur Respir J. 2024 Apr 4;63(4):2302031. doi: 10.1183/13993003.02031-2023 (PMC10991834; doi:10.1183/13993003.02031-2023)
Supplement: Supplementary file 1 [file ERJ-02031-2023.Shareable.pdf]

EUROPEAN RESPIRATORY JOURNAL
RESEARCH LETTER
B. WOOLF ET AL.

SHAREABLE PDF

Multi-biobank summary data Mendelian randomisation does not support a causal effect of IL-6 signalling on risk of pulmonary arterial hypertension

Benjamin Woolf [1,2,3], James A. Perry [4], Charles C. Hong [5], Martin R. Wilkins [6], Mark Toshner [7], Dipender Gill [8], Stephen Burgess [3,9] and Christopher J. Rhodes [6]

[1]The MRC Integrative Epidemiology Unit, University of Bristol, Bristol, UK. [2]School of Psychological Science, University of Bristol, Bristol, UK. [3]The MRC Biostatistics Unit, University of Cambridge, Cambridge, UK. [4]Department of Medicine, University of Maryland School of Medicine, Baltimore, MD, USA. [5]Department of Medicine, Michigan State University College of Human Medicine, East Lansing, MI, USA. [6]National Heart and Lung Institute, Imperial College London, London, UK. [7]Department of Medicine, University of Cambridge, Cambridge, UK. [8]Department of Epidemiology and Biostatistics, School of Public Health, Imperial College London, London, UK. [9]British Heart Foundation Cardiovascular Epidemiology Unit, Department of Public Health and Primary Care, University of Cambridge, Cambridge, UK.

Corresponding author: Benjamin Woolf (benjamin.woolf@bristol.ac.uk)

Check for updates

Shareable abstract (@ERSpublications)
In the most comprehensive analysis to date, this study failed to detect an association of genetically predicted CRP-weighted IL-6 signalling or CRP-weighted IL-6R signalling with PAH risk using all available PAH GWAS data https://bit.ly/3T5h5uj

Cite this article as: Woolf B, Perry JA, Hong CC, *et al*. Multi-biobank summary data Mendelian randomisation does not support a causal effect of IL-6 signalling on risk of pulmonary arterial hypertension. *Eur Respir J* 2024; 63: 2302031 [DOI: 10.1183/13993003.02031-2023].

This extracted version can be shared freely online.

Copyright ©The authors 2024.

This version is distributed under the terms of the Creative Commons Attribution Licence 4.0.

Received: 15 Nov 2023
Accepted: 14 Feb 2024

To the Editor:

Interleukin (IL)-6 has been linked with the pathobiology of pulmonary arterial hypertension (PAH). IL-6 plasma levels are elevated in PAH patients and closely linked to survival [1]. Both increased IL-6 activity and gene knockout influence the development of, and resistance to, pulmonary hypertension in animal models [2–4]. IL-6 can repress expression of *BMPR2*, a gene key in PAH risk [5].
